# In-Hospital Delirium and Disability and Cognitive Impairment After COVID-19 Hospitalization

**DOI:** 10.1001/jamanetworkopen.2024.19640

**Published:** 2024-07-02

**Authors:** Ramya Kaushik, Gail J. McAvay, Terrence E. Murphy, Denise Acampora, Katy Araujo, Peter Charpentier, Sumon Chattopadhyay, Mary Geda, Thomas M. Gill, Tamar A. Kaminski, Seohyuk Lee, Judy Li, Andrew B. Cohen, Alexandra M. Hajduk, Lauren E. Ferrante

**Affiliations:** 1 Department of Internal Medicine, Yale School of Medicine, New Haven, Connecticut; 2 Section of Geriatrics, Department of Internal Medicine, Yale School of Medicine, New Haven, Connecticut; 3 Department of Public Health Sciences, Penn State College of Medicine, Hershey, Pennsylvania; 4 CRI Web Tools, Durham, Connecticut; 5 Clinical and Translational Science Institute, University of Utah, Salt Lake City; 6 Yale School of Medicine, New Haven, Connecticut; 7 Section of Pulmonary Critical Care, and Sleep Medicine, Department of Internal Medicine, Yale School of Medicine, New Haven, Connecticut

## Abstract

**Importance:**

Older adults who are hospitalized for COVID-19 are at risk of delirium. Little is known about the association of in-hospital delirium with functional and cognitive outcomes among older adults who have survived a COVID-19 hospitalization.

**Objective:**

To evaluate the association of delirium with functional disability and cognitive impairment over the 6 months after discharge among older adults hospitalized with COVID-19.

**Design, Setting, and Participants:**

This prospective cohort study involved patients aged 60 years or older who were hospitalized with COVID-19 between June 18, 2020, and June 30, 2021, at 5 hospitals in a major tertiary care system in the US. Follow-up occurred through January 11, 2022. Data analysis was performed from December 2022 to February 2024.

**Exposure:**

Delirium during the COVID-19 hospitalization was assessed using the Chart-based Delirium Identification Instrument (CHART-DEL) and CHART-DEL-ICU.

**Main Outcomes and Measures:**

Primary outcomes were disability in 15 functional activities and the presence of cognitive impairment (defined as Montreal Cognitive Assessment score <22) at 1, 3, and 6 months after hospital discharge. The associations of in-hospital delirium with functional disability and cognitive impairment were evaluated using zero-inflated negative binominal and logistic regression models, respectively, with adjustment for age, month of follow-up, and baseline (before COVID-19) measures of the respective outcome.

**Results:**

The cohort included 311 older adults (mean [SD] age, 71.3 [8.5] years; 163 female [52.4%]) who survived COVID-19 hospitalization. In the functional disability sample of 311 participants, 49 participants (15.8%) experienced in-hospital delirium. In the cognition sample of 271 participants, 31 (11.4%) experienced in-hospital delirium. In-hospital delirium was associated with both increased functional disability (rate ratio, 1.32; 95% CI, 1.05-1.66) and increased cognitive impairment (odds ratio, 2.48; 95% CI, 1.38-4.82) over the 6 months after discharge from the COVID-19 hospitalization.

**Conclusions and Relevance:**

In this cohort study of 311 hospitalized older adults with COVID-19, in-hospital delirium was associated with increased functional disability and cognitive impairment over the 6 months following discharge. Older survivors of a COVID-19 hospitalization who experience in-hospital delirium should be assessed for disability and cognitive impairment during postdischarge follow-up.

## Key Points

**Question:**

Among older adults (aged 60 years or older) hospitalized for COVID-19, is in-hospital delirium associated with functional disability and cognitive impairment over the 6 months after discharge?

**Findings:**

In this cohort study of 311 older adults hospitalized for acute COVID-19 illness, in-hospital delirium was associated with both functional disability and cognitive impairment over the 6 months after hospital discharge.

**Meaning:**

These findings suggest that after discharge from hospitalization for COVID-19, older adults who experience in-hospital delirium may need to be counseled, screened, and provided resources to mitigate their risk of long-term functional disability and cognitive impairment.

## Teaser

This cohort study examines the association of in-hospital delirium with functional disability and cognitive impairment for the first 6 months after discharge among older adults (aged 60 years or older) hospitalized with COVID-19.

## Introduction

Older adults have a higher likelihood of experiencing severe illness due to COVID-19 and are more likely than younger individuals to require hospitalization and intensive care unit (ICU) admission.^1^ Among older adults hospitalized for COVID-19, delirium is a commonly observed condition, with one study^2^ reporting it as the sixth most frequent symptom in older adults presenting to the emergency department. Studies^3,4,5^ on COVID-19 estimate that delirium rates range from 11% to 65% in hospitalized patients.

Delirium is a potent risk factor for hospital-related complications, including longer hospitalizations, unplanned ICU admission, discharge to a nursing facility, and all-cause mortality.^6,7,8^ It has long-term adverse sequelae and has been associated with persistent cognitive decline and institutionalization.^9,10,11^ The COVID-19 pandemic amplified certain factors associated with increased risk of delirium. These include prolonged hospital stays, the administration of medications such as sedatives, social isolation resulting from infection control measures, immobility, and communication barriers between patients and health care practitioners arising from the use of personal protective equipment.^12,13^ Staff redeployment and visitation restrictions during the pandemic also disrupted established strategies to prevent and treat delirium. These included frequent attention from staff or trained volunteers to reorient patients, as well as visits from family and friends.

It is crucial, in this context, to understand the repercussions of delirium during hospitalization with COVID-19, including the relationship between delirium and the outcomes that matter most to older persons. In this study, we sought to evaluate the association of in-hospital delirium with functional and cognitive impairment over the 6-month period following discharge.

## Methods

### Study Population

Participants were drawn from the VALIANT (COVID-19 in Older Adults: A Longitudinal Assessment) cohort,^14^ which was designed to evaluate patient-centered outcomes among older survivors of a COVID-19 hospitalization. The VALIANT study is a prospective cohort study of community-living older adults who were hospitalized with COVID-19 at 5 hospitals within the Yale-New Haven Health System, a major tertiary care system. Participants were eligible if they were at least 60 years old, spoke English or Spanish, and had a polymerase chain reaction–confirmed diagnosis of SARS-CoV-2 infection either during or immediately before hospital admission. Participants were not eligible if they had advanced dementia, were long-term residents at a skilled nursing facility, or were transitioning to hospice. Verbal informed consent was obtained via telephone or video conferencing software by trained research coordinators. If there was concern about a participant’s decision-making ability, we conducted the University of San Diego Brief Assessment for Capacity to Consent.^15^ If decisional impairment was confirmed, proxies were sought for informed consent, with verbal assent from participants.

Participants were enrolled between June 18, 2020, and June 30, 2021, with follow-up occurring through January 11, 2022. Information about participants’ physical function, cognition, and symptoms were collected at the baseline interview, which took place during or within 2 weeks of the index hospitalization, and subsequently at follow-up interviews at 1, 3, and 6 months after hospital discharge, as described elsewhere.^14^ All assessments were conducted remotely, either via telephone or videoconference. The follow-up rate in the VALIANT cohort was 92.7%. This research was approved by Yale University institutional review board and was developed according to the Strengthening the Reporting of Observational Studies in Epidemiology (STROBE) reporting guidelines for cohort studies.^16^

### Baseline Characteristics and Hospital Data

Participant demographics, including sex, race, and ethnicity, were abstracted from the electronic medical record (EMR). Data on race and ethnicity were included in this study because the COVID-19 pandemic disproportionately affected individuals from minoritized racial and ethnic groups. As described elsewhere,^14^ the baseline interview included an assessment of prehospitalization functional status (1 month before hospital admission), which could be completed by a proxy if necessary. It is well-established that retrospective reports of prehospital functional status by hospitalized older adults and their surrogates are valid and reliable.^17^ Because it was not possible to conduct formal cognitive assessments before study enrollment during the COVID-19 hospitalization, preexisting cognitive impairment over the 3 years before the index hospitalization was abstracted from the EMR. Abstractions were done by trained research staff (A.M.H., J.L., and A.B.C.) using a standard adjudication guide developed using previously developed criteria,^18^ and double adjudication was completed on 20% of cases to ensure quality control. Details about the index hospitalization, including severity of illness, Sequential Organ Failure Assessment score,^19^ and need for advanced respiratory support were obtained from Yale Department of Medicine COVID-19 Data Explorer.^20^

### Assessment of Functional Disability

Disability was assessed in 15 functional activities, including 7 basic activities of daily living (bathing, dressing, transferring from a chair, walking inside the house, eating, toileting, and grooming),^21^ 5 instrumental activities of daily living (shopping, housework, meal preparation, taking medication, and managing finances),^22^ and 3 mobility activities (walking a quarter mile, climbing a flight of stairs, and lifting or carrying 10 pounds) at 1, 3, and 6 months following hospitalization.^23^ As in prior work,^24,25^ disability was defined as being unable to complete the task or needing assistance from another person to complete the task.

### Assessment of Cognition

Cognition was evaluated using the Montreal Cognitive Assessment (MoCA) 5-minute protocol at 1, 3, and 6 months after hospital discharge.^26^ This tool has been adapted to be used over the telephone, and it measures attention, orientation, language, verbal learning and memory, and executive function. A score of less than 22 (range, 0 to 30) suggests cognitive impairment.^27^ Participants, but not proxies, could complete the MoCA.

### Assessment of In-Hospital Delirium Status

The presence or absence of delirium during the COVID-19 hospitalization was assessed using the validated Chart-Based Delirium Identification Instruments (CHART-DEL^28^ and CHART-DEL ICU^29^). These methods for delirium detection involve reviewing a patient’s medical record for diagnoses synonymous with delirium or a change to patient’s baseline mental status as indicated by trigger words or phrases that are associated with high positive predictive values for delirium. Research personnel (J.L. and T.A.K.) reviewed notes from each day of the hospitalization and used structured rubrics to record whether delirium was present at any time and to rate their confidence in this assessment (definite, probable, possible, uncertain, or no evidence). To maximize sensitivity, we used a CHART-DEL cutoff where delirium was considered present at any confidence level other than no evidence.^28^ J.L and T.A.K. were blinded to the primary outcomes of this study and did not participate in the analysis.

### Covariate Selection

Both models were adjusted for age and month of follow-up, given the longitudinal nature of the analysis. The models were also adjusted for the baseline measure of the respective outcome: preadmission disability (1 month before admission) or preexisting cognitive impairment, as already described.

### Assembly of the Analytic Sample

Assembly of the analytic samples is presented in eFigure in Supplement 1. The analytic sample for functional disability consisted of participants who had baseline measures and at least 1 follow-up interview. The analytic sample for cognition included participants who completed at least 1 follow-up MoCA and had a measure of baseline cognitive function. Participants who were missing data on preexisting cognitive function (8 participants), had follow-up interviews that were all completed by proxy (15 participants), or were completely missing the MoCA at all follow-up interviews (17 participants) were excluded.

### Statistical Analysis

Data analysis was performed from December 2022 to February 2024. Baseline characteristics of participants were summarized by in-hospital delirium status, using frequencies and percentages for categorical variables or means and SDs for continuous measures. We determined the mean count of functional disability and the percentage of participants with cognitive impairment at each follow-up visit.

Missingness in the functional outcome measure is presented in eTable 1 in Supplement 1. There was partial missingness in 2.4% of the disability outcome measures (ranging from 1.3% at baseline to 4.9% at month 3). Missingness in the cognitive impairment outcome is presented in eTable 2 in Supplement 1. If a MoCA was completely missing at any follow-up interview, it was excluded; we did not multiply impute completely missing MoCAs because we did not think these could be missing at random. If the MoCA was partially complete, the missing scale items were thought to be missing at random and could be imputed. There was partial missingness in 2.8% of MoCA outcome measures. Missingness in covariates was very low (<2%). We used multiple imputation methods based on fully conditional specifications to generate 30 imputations, as implemented in PROC MI in SAS statistical software version 9.4 (SAS Institute).^30^

The longitudinal associations between the exposure of in-hospital delirium and the outcomes of count of functional disability and presence of cognitive impairment were evaluated using multivariable zero-inflated negative binominal and multivariable logistic regression, respectively. All models included the covariates of age, month of follow-up, and the baseline value of each respective outcome. The death rate during follow-up was low (1.9%), so no sensitivity analyses for the competing risk of death were performed. Three patients died before the 3-month interview and an additional 3 died before the 6-month interview.

For the count of functional disability, we used the zinb command in Stata statistical software version 15.1 (StataCorp)^31^ for zero-inflated negative binomial modeling that accounts for the dependence of observations within subjects via a robust cluster estimator of the SE. For the cognitive impairment outcome, a generalized estimating equation logistic regression model with an autoregressive correlation structure was estimated using PROC GENMOD in SAS.^30^ Multivariable models were separately fit to each of the imputed datasets and the 30 sets of estimated coefficients were combined using Rubin rules. Statistical significance was defined as a 2-sided *P* < .05.

## Results

Descriptive characteristics stratified by in-hospital delirium status for each analytic cohort are presented in Table 1, and characteristics of the overall function cohort are presented in eTable 3 in Supplement 1. The mean (SD) age of the functional disability sample of 311 patients was 71.3 (8.5) years, and 163 (52.4%) were female. More than one-third of participants were individuals from minoritized racial and ethnic groups; 1 patient (0.3%) was Asian, 1 (0.3%) was Black Hispanic, 72 (23.2%) were Black non-Hispanic, 33 (10.6%) were Hispanic (race not reported), 1 (0.3%) was non-Hispanic (race not reported), 5 (1.6%) were White Hispanic, 197 (63.3%) were White non-Hispanic, and 1 (0.3%) was of unknown race and ethnicity. Among participants in the functional disability sample, 99 (31.8%) had Medicaid insurance. There were 271 participants in the cognition sample. In the functional disability sample of 311 participants, 49 (15.8%) participants experienced in-hospital delirium. In the cognition sample of 271 participants, 31 (11.4%) experienced in-hospital delirium. Participants with in-hospital delirium were older than those without delirium in both the function sample (mean [SD] age, 75.6 [8.5] years vs 70.5 [8.3] years) and the cognition sample (mean [SD] age, 73.1 [7.4] years vs 70.2 [8.0] years). Those with in-hospital delirium also had greater preexisting disability in functional activities and prehospitalization cognitive impairment compared with those who did not develop in-hospital delirium. In both samples, a higher proportion of participants with in-hospital delirium were admitted to the stepdown unit or ICU and required mechanical ventilation (eTable 4 in Supplement 1). In the functional disability cohort, 29 participants (9.3%) were admitted to the ICU. The 6-month follow-up rate in our analytic sample was 96.4%, reflecting 294 interviews of 305 eligible for completion.

**Table 1.** Baseline Characteristics of Participants in Each Analytic Sample (Functional Disability and Cognition), by Presence of In-Hospital Delirium

Characteristic^a^	Participants, No. (%)			
	Functional disability (n = 311)		Cognition (n = 271)	
	Delirium (n = 49)	No delirium (n = 262)	Delirium (n = 31)	No delirium (n = 240)
Age, mean (SD), y	75.6 (8.5)	70.5 (8.3)	73.1 (7.4)	70.2 (8.0)
Sex				
Female	25 (51.0)	138 (52.7)	18 (58.1)	130 (54.2)
Male	24 (49.0)	124 (47.3)	13 (41.9)	110 (45.8)
Race and ethnicity				
Asian, non-Hispanic	0	1 (0.4)	0	1 (0.4)
Black, Hispanic	1 (2.0)	0	1 (3.2)	0
Black, non-Hispanic	11 (22.5)	61 (23.3)	9 (29.0)	53 (22.1)
Hispanic, race not reported	5 (10.2)	28 (10.7)	3 (9.7)	24 (10.0)
Non-Hispanic, race not reported	0	1 (0.4)	0	1 (0.4)
White, Hispanic	0	5 (1.9)	0	4 (1.7)
White, non-Hispanic	32 (65.3)	165 (63.0)	18 (58.1)	156 (65.0)
Unknown race and ethnicity	0	1 (0.4)	0	1 (0.4)
Medicaid	18 (36.7)	81 (30.9)	13 (41.9)	73 (30.4)
Marital status				
Married or living with partner	22 (44.9)	123 (47.1)	13 (41.9)	114 (47.7)
Divorced, separated, or widowed	19 (38.8)	90 (34.5)	11 (35.5)	83 (34.7)
Single	7 (14.3)	48 (18.4)	7 (22.6)	42 (17.6)
Other	1 (2.0)	0	0	0
Living alone	6 (12.5)	81 (30.9)	4 (13.3)	73 (30.4)
Comorbidity count (range, 0-10), median (IQR)^b^	3 (2-5)	2 (1-4)	3 (2-5)	2 (1-4)
No. of functional activities with experienced disability (range, 0-15), median (IQR)^c^	2 (0-7)	0 (0-3)	0.5 (0-3)	0 (0-2)
Prehospitalization cognitive impairment	12 (26.1)	11 (4.3)	3 (9.7)	7 (2.9)

^a^ Numbers may not sum to the column total because of missing covariates.

^b^ Comorbidities included hypertension, myocardial infarction, heart failure, cerebrovascular disease (stroke, transient ischemic attack, or intracranial hemorrhage), diabetes, chronic lung disease, chronic kidney disease, end-stage kidney disease, liver disease, immunocompromised status (autoimmune disease, HIV positive, or receipt of solid-organ transplant), and malignant entity (solid tumors, leukemia or lymphoma, or metastatic disease).

^c^ Tasks included 7 basic activities of daily living (eating, dressing, bathing, toileting, grooming, getting in and out of a chair, and walking around indoors), 5 instrumental activities of daily living (doing housework, going shopping, preparing a meal, taking medications, and managing finances), and 3 mobility activities (walking a quarter of a mile, climbing stairs, and lifting or carrying heavy objects). A higher score indicates greater disability.

### Delirium and Functional Disability Over the 6 Months After the COVID-19 Hospitalization

The unadjusted mean disability count before hospitalization was 4.0 disabilities (95% CI, 2.5-5.4 disabilities) among participants who experienced in-hospital delirium. It increased to 6.6 disabilities (95% CI, 4.9-8.2 disabilities) at 1 month after hospital discharge and was 5.3 disabilities (95% CI, 3.7-6.9 disabilities) at 6 months after hospital discharge (Figure). The unadjusted mean disability count before hospitalization was 1.8 disabilities (95% CI, 1.5-2.2 disabilities) in participants without in-hospital delirium. It increased to 2.7 disabilities (95% CI, 2.2-3.1 disabilities) at 1-month follow-up and was 2.1 disabilities (95% CI, 1.7-2.5 disabilities) at 6-month follow-up. In the multivariable model, in-hospital delirium was found to be associated with an increased disability count over the 6 months after a COVID-19 hospitalization (rate ratio, 1.32; 95% CI, 1.05-1.66).

**Figure.**
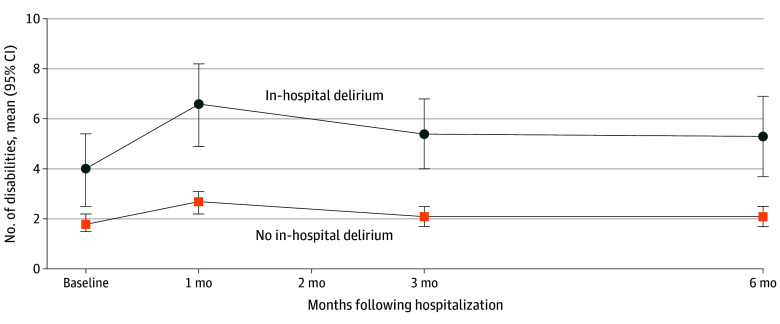
Disability Count After COVID-19 Hospitalization by Status of In-Hospital Delirium Graph shows mean unadjusted disability count at 1, 3, and 6 months after COVID-19 hospitalization, stratified by presence of in-hospital delirium; error bars denote 95% CIs. Baseline disability count is included as a reference point. Disability in 7 basic activities of daily living, 5 instrumental activities of daily living, and 3 mobility activities (range, 0-15 activities) was assessed at each interview.

### Delirium and Cognitive Impairment Over the 6 Months After a COVID-19 Hospitalization

The unadjusted percentage of participants with cognitive impairment before hospitalization was 9.7% (3 of 31 participants) among participants who experienced in-hospital delirium and 2.9% (7 of 240 participants) among participants without in-hospital delirium (Table 2). At 1-month follow-up, 12 participants (63.6%) with in-hospital delirium experienced cognitive impairment compared with 49 participants (22.6%) without in-hospital delirium. The proportion of participants with cognitive impairment at the 3-month follow-up were similar in the 2 groups, approximately 14% for both. Of note, however, there was more missingness in the 3-month follow-up interviews among participants who had exhibited cognitive impairment than those not impaired at the 1-month interview. At 6-month follow-up, 8 participants (34.8%) with delirium had cognitive impairment, compared with 43 participants (20.7%) without delirium. In the multivariable analysis, in-hospital delirium was associated with cognitive impairment over the 6 months after a COVID-19 hospitalization (odds ratio, 2.48; 95% CI, 1.28-4.82).

**Table 2.** Participants With Cognitive Impairment After COVID-19 Hospitalization, by Presence of In-Hospital Delirium

Interview time (No. of participants)	Participants with cognitive impairment, No./total No. (%)	
	In-hospital delirium	No in-hospital delirium
Baseline (n = 271)^a^	3/31 (9.7)	7/240 (2.9)
1 mo (n = 236)^b^	12/19 (63.2)	49/217 (22.6)
3 mo (n = 222)^b^	3/22 (13.6)	29/200 (14.5)
6 mo (n = 231)^b^	8/23 (34.8)	43/208 (20.7)

^a^ Baseline cognitive impairment, included here for reference, was abstracted from the electronic medical record by abstractors blinded to the study outcomes with double adjudication in 20% of the cases.

^b^ Cognitive status at 1, 3, and 6 months was evaluated using the Montreal Cognitive Assessment 5-minute protocol, with a score less than 22 (range, 0-30) suggesting cognitive impairment.

## Discussion

In this cohort study, we evaluated longitudinal functional and cognitive outcomes among older adults with COVID-19 who experienced in-hospital delirium. We found that in-hospital delirium during a COVID-19 hospitalization was associated with increased rates of functional disability and cognitive impairment over the 6 months after discharge.

Delirium has been recognized as a severe complication of COVID-19 and has been linked to worse outcomes, including increased in-hospital mortality, ICU admission, length of stay, and in-hospital complications.^32,33,34,35^ In our functional disability sample, delirium was present in 15.8% of participants, which corroborates previous studies^3,4,5,36^ that reported a wide range overall incidence of delirium, from 11% to 65%, among patients hospitalized with COVID-19. In our study, only 9.3% of participants were admitted to the ICU, which might account for the lower reported incidence of delirium. The incidence of delirium was much higher among participants who were admitted to the ICU than those who were not in the ICU.

Our study builds on previous work evaluating the association between delirium and functional and cognitive outcomes. Prior work^37^ found that delirium was associated with poor short-term functional outcomes, but not cognitive outcomes, 4 weeks following COVID-19 hospitalization. However, that study^37^ was smaller (71 participants) than the present study and did not focus on older adults. Other studies have examined long-term cognitive and functional impairment at 3 months,^38^ 6 months,^39,40,41,42^ and 12 months^41,43^ following illness with COVID-19, without investigating the association with delirium. To our knowledge, our study is the first to demonstrate the association of in-hospital delirium with disability and cognitive impairment among older survivors of a COVID-19 hospitalization.

Studies^7,9,11,44^ conducted before the COVID-19 pandemic found that delirium is a well-established complication for hospitalized older adults and is associated with long-term cognitive and functional impairment. Prior work^43,44,45,46^ has highlighted multiple factors predisposing older adults to delirium, including preexisting cognitive or functional disability, vision or hearing impairment, multimorbidity, and polypharmacy. Evidence-based programs such as the Hospital Elder Life Program (HELP) have been shown to be a feasible and effective method to reduce in-hospital delirium with use of strategies including reorientation, nonpharmacologic sleep protocols, early mobilization, use of hearing and visual aids, and identification and treatment of dehydration.^47,48^ Unfortunately, a lack of staff, use of personal protective equipment, enforcement of isolation policies with visitor restrictions, and increased use of sedative medications worsened recognition and treatment of delirium during the COVID-19 pandemic.^12^ Our findings suggest that a renewed focus on implementation of these evidence-based interventions is needed to prevent vulnerable older adults from developing delirium during hospitalization with COVID-19. Although these strategies may not have been feasible during the peak of the pandemic, older adults are continuing to be hospitalized with COVID-19 infection, and it will be important for health care personnel to adapt and follow the HELP paradigm to prevent delirium.^49^ With the return of normal visitation policies compared with early in the pandemic, the inclusion of family member participation for delirium prevention efforts in the hospital setting, with programs such as Family-Augmented-HELP, may enhance delirium prevention efforts.^50^ Moreover, health care personnel who care for older adults after discharge should recognize that older COVID-19 survivors who experience in-hospital delirium are at increased risk of long-term cognitive impairment and functional disability. Newer studies^51,52,53^ suggest potential interventions for cognitive and functional impairment following COVID-19 including cognitive rehabilitation and exercises focused on balance, endurance, and resistance training.

### Strengths and Limitations

A major strength of this study is the high follow-up rate in both the parent cohort and among participants in the current study. This facilitated the rigorous evaluation of physical and cognitive function over time. An additional strength is the inclusion of baseline functional and cognitive measures prior to hospitalization, which provide important context for understanding longitudinal outcomes after COVID-19 and were included in our multivariable models. Another strength was our assessment of delirium using validated methods (CHART-DEL and CHART-DEL ICU) where the abstractors were blinded to the study outcomes. Finally, the study participants were racially, ethnically, and socioeconomically diverse, with 35.7% of Black race and/or Hispanic ethnicity and 31.8% covered by Medicaid.

Our study also had limitations. Baseline cognitive function could not be formally assessed before the COVID-19 hospitalization, so we used an EMR review method to abstract preexisting cognitive impairment up to 3 years before admission. Although the EMR review was performed by 3 authors blinded to the study outcome with double adjudication, we would not have been able to detect preexisting cognitive impairment that had not been documented in the EMR. However, a majority of participants had previous encounters with the health system, including EMR messages, telephone encounters, outpatient visits, emergency department visits, and prior hospitalizations. Within these encounters, many participants had undergone formal cognitive assessments or a review of systems in which memory or cognitive impairment was documented, which provided valuable information for determining baseline cognitive status. In addition, it was difficult to enroll the most severely ill patients because they could not provide consent and proxies were not present in the hospital, so the cohort may be slightly biased toward a healthier population. However, estimates of delirium in our cohort are within the range seen in other cohorts. This study was also conducted before widespread administration of COVID-19 vaccines, and our findings may not fully reflect outcomes among vaccinated patients hospitalized in the current era. However, it is important to note that older adults continue to be hospitalized for COVID-19, highlighting the ongoing relevance of our work. In addition, our study follow-up period was 6 months, and longer term follow-up would provide additional information about the trajectory of cognitive and functional outcomes following COVID-19 hospitalization. Furthermore, the number of interviews was lowest at 3-month follow-up, which may have resulted in lower estimates of cognitive impairment at that time point.

## Conclusions

In summary, among older adults hospitalized for COVID-19, in-hospital delirium was associated with increased functional disability and cognitive impairment over the 6 months following discharge. Future studies should evaluate whether delirium prevention strategies and cognitive screening within the hospital, as well as rehabilitation following discharge, can effectively reduce the deleterious sequelae of in-hospital delirium among older persons hospitalized for COVID-19.

## Notes

Supplement 1. **eFigure.** Flow diagram for assembly of the analytic sample for both outcomes **eTable 1.** Missingness in 15-item count of disabilities (functional outcome) at each timepoint **eTable 2.** Complete and partial missingness of MoCA (cognitive outcome) at each timepoint **eTable 3.** Characteristics of entire cohort at baseline, during hospitalization, and following hospitalization **eTable 4.** Hospital characteristics of each analytic group by delirium status

Supplement 2.
Data Sharing Statement
