# Supplementary material for: In-Hospital Delirium and Disability and Cognitive Impairment After COVID-19 Hospitalization
Source: JAMA Netw Open. 2024 Jul 2;7(7):e2419640. doi: 10.1001/jamanetworkopen.2024.19640 (PMC11220565; doi:10.1001/jamanetworkopen.2024.19640)
Supplement: Supplement 1. — eFigure. Flow diagram for assembly of the analytic sample for both outcomes eTable 1. Missingness in 15-item count of disabilities (functional outcome) at each timepoint eTable 2. Complete and partial missingness of MoCA (cognitive outcome) at each timepoint eTable 3. Characteristics of entire cohort at baseline, during hospitalization, and following hospitalization eTable 4. Hospital characteristics of each analytic group by delirium status [file jamanetwopen-e2419640-s001.pdf]

Supplemental Online Content

Kaushik R, McAvay GJ, Murphy TE, et al. In-hospital delirium and disability and cognitive impairment after COVID-19 hospitalization. *JAMA Netw Open*. 2024;7(7):e2419640. doi:10.1001/jamanetworkopen.2024.19640

eFigure. Flow diagram for assembly of the analytic sample for both outcomes
eTable 1. Missingness in 15-item count of disabilities (functional outcome) at each timepoint
eTable 2. Complete and partial missingness of MoCA (cognitive outcome) at each timepoint
eTable 3. Characteristics of entire cohort at baseline, during hospitalization, and following hospitalization
eTable 4. Hospital characteristics of each analytic group by delirium status

This supplemental material has been provided by the authors to give readers additional information about their work.

© 2024 Kaushik R et al. *JAMA Network Open.*

eFigure: Flow diagram for assembly of the analytic sample for both outcomes.

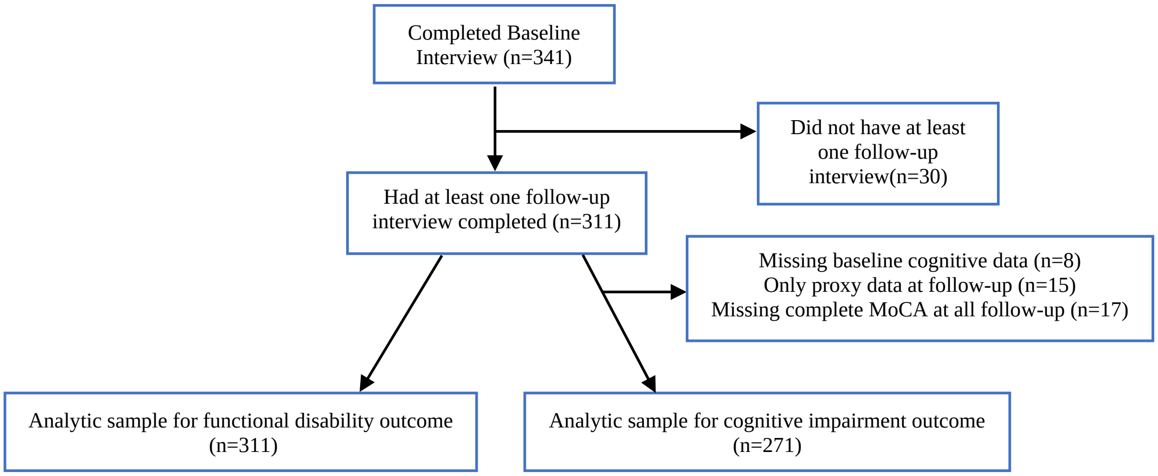

For the function analytic sample, participants needed to have at least one follow-up interview (n=311). For the cognitive analytic sample, participants were excluded if missing baseline cognitive data (n=8), only proxy completed follow-up interviews were done (n=15), or if complete MoCA assessments were missing at all follow-up time-points (n=17). This resulted in an analytic sample of 271 for cognition.

© 2024 Kaushik R et al. *JAMA Network Open.*

eTable 1. Missingness in 15-item count of disabilities (functional outcome) at each timepoint

Baseline		One Month		Three Months		Six Months	
Number of Items Missing	n	Number of Items Missing	n	Number of Items Missing	n	Number of Items Missing	n
0	307	0	270	0	272	0	289
1	2	1	11	1	4	1	4
9	1	3	1	12	1	8	1
14	1	6	1				
		12	1				

There was partial missingness in 2.4% of the disability outcome measures, ranging from 1.3% at baseline to 4.9% at one month. Items were multiply imputed if partially missing.

© 2024 Kaushik R et al. *JAMA Network Open.*

eTable 2: Complete and partial missingness of MoCA (cognitive outcome) at each timepoint

Missingness	One Month (n)	Three Months (n)	Six Months (n)
None	236	222	231
Partial	9	6	5
Complete	7	18	20

The table presents the number of participants with partial or complete missingness of the MoCA at each timepoint. If the MoCA was completely missing at any follow-up interview, it was excluded from the analysis. Of these excluded MoCA assessments (bottom row of table), some were completely missing due to proxy completion of the follow-up interview: 1 month (n=2), 3 months (n=2), 6 months (n=1). If MoCA was partially completed, missing scale items were multiply imputed. There was partial missingness in 2.8% of MoCA outcome measures, ranging from 2.1% at month 6 to 3.7% at month 1.

© 2024 Kaushik R et al. *JAMA Network Open.*

eTable 3: Characteristics of the study cohort at baseline, during hospitalization, and following hospitalization

**Characteristic[a]**	**All (n=311)**
Age in years, mean (SD)	71.3 (8.5)
Female sex, n (%)	163 (52.4)
Male sex, n (%)	148 (47.6)
Race and ethnicity, n (%)	
Asian, non-Hispanic	1 (0.3)
Black, Hispanic	1 (0.3)
Black, non-Hispanic	72 (23.2)
Hispanic, race not reported	33 (10.6)
Non-Hispanic, race not reported	1 (0.3)
White, Hispanic	5 (1.6)
White, non-Hispanic	197 (63.3)
Unknown race and ethnicity	1 (0.3)
Medicaid, n(%)	99 (31.8)
Marital status:	
Married or living with partner	145 (46.8)
Divorced, separated or widowed	109 (35.2)
Single	55 (17.7)
Other	1 (0.3)
Living alone, n (%)	87 (28.1)
Comorbidity count (range, 0-10), median (IQR)[b]	3 (1-4)
# of functional activities with experienced disability (range, 0-15), median (IQR)[c]	0 (0-3)
Pre-hospitalization cognitive impairment	23 (7.6)
Highest level of care	
Floor	256 (82.3)
Stepdown unit	26 (8.4)
Intensive Care Unit	29 (9.3)
Highest level of Oxygen Support, n (%)	
None	12 (3.9)
Nasal cannula	251 (80.7)
High flow oxygen	30 (9.7)

© 2024 Kaushik R et al. *JAMA Network Open.*

Non-invasive positive pressure ventilation	10 (3.2)
Mechanical ventilation	8 (2.6)
SOFA score, median (IQR)[d]	3 (2-4)
Discharge destination, n (%)	
Home	167 (53.7)
Home with services	99 (31.8)
Skilled nursing facility	41 (13.2)
Long-term acute care facility	2 (0.6)
Left against medical advice	2 (0.6)

[a]Numbers may not sum to the column total due to missing covariates.
[b]Comorbidities included hypertension, myocardial infarction, heart failure, cerebrovascular disease (stroke, transient ischemic attack, or intracranial hemorrhage), diabetes mellitus, chronic lung disease, chronic kidney disease, end-stage renal disease, liver disease, immunocompromised status (autoimmune disease, HIV positive, or receipt of solid organ transplant), and malignancy (solid tumors, leukemia or lymphoma, or metastatic disease).
[c]Tasks included seven basic activities of daily living (eating, dressing, bathing, toileting, grooming, getting in and out of a chair, walking around indoors); five instrumental activities of daily living (doing housework, going shopping, preparing a meal, taking medications, managing finances); and three mobility activities (walking a quarter of a mile, climbing stairs, and lifting or carrying heavy objects). A higher score indicates greater disability.
[d]SOFA = Sequential Organ Failure Assessment. The SOFA score is based on oxygenation, platelet count, Glasgow Coma Scale, bilirubin, mean arterial pressure or receipt of vasoactive agents (dopamine, dobutamine, epinephrine, or norepinephrine), and creatinine. Scores range from 0-24, where higher scores indicate worse severity of illness.

© 2024 Kaushik R et al. *JAMA Network Open.*

eTable 4. Hospital characteristics of each analytic group by delirium status.

Characteristics	Functional Disability (n=311)		Cognition (n=271)	
	**Delirium (n=49)**	**No delirium (n=262)**	**Delirium (n=31)**	**No delirium (n=240)**
Hospital Highest level of care				
Floor	27 (55.1)	229 (87.4)	17 (54.8)	210 (87.5)
Stepdown unit	7 (14.3)	19 (7.3)	4 (12.9)	17 (7.1)
Intensive Care Unit	15 (30.6)	14 (5.3)	10 (32.3)	13 (5.4)
Highest level of Oxygen Support, n (%)				
None	0 (0.0)	12 (4.6)	0 (0.0)	10 (4.2)
Nasal cannula	31 (63.3)	220 (84.0)	19 (61.3)	203 (84.6)
High flow oxygen	7 (14.3)	23 (8.8)	4 (12.9)	21 (8.7)
Non-invasive positive pressure ventilation	3 (6.1)	7 (2.7)	1 (3.2)	6 (2.5)
Mechanical ventilation	8 (16.3)	0 (0.0)	7 (22.6)	0 (0.0)
SOFA score, maximum (range, 0-24), median (IQR)[a]	5 (2,8)	2.5 (2,4)	5 (2,9)	2 (2,3)
Discharge destination, n (%)				
Home	13 (26.5)	54 (58.8)	11 (35.5)	144 (60.0)
Home with services	19 (38.8)	80 (30.5)	12 (38.7)	72 (30.0)
Skilled nursing facility	16 (32.7)	25 (9.5)	7 (22.6)	21 (8.8)
Long-term acute care facility	1 (2.0)	1 (0.4)	1 (3.2)	1 (0.4)
Left against medical advice	0 (0.0)	2 (0.8)	0 (0.0)	2 (0.8)

[a]SOFA = Sequential Organ Failure Assessment. The SOFA score is based on oxygenation, platelet count, Glasgow Coma Scale, bilirubin, mean arterial pressure or receipt of vasoactive agents (dopamine, dobutamine, epinephrine, or norepinephrine), and creatinine. Scores range from 0-24, where higher scores indicate worse severity of illness.

© 2024 Kaushik R et al. *JAMA Network Open.*
