# Supplementary material for: In-Hospital Delirium and Disability and Cognitive Impairment After COVID-19 Hospitalization
Source: JAMA Netw Open. 2024 Jul 2;7(7):e2419640. doi: 10.1001/jamanetworkopen.2024.19640 (PMC11220565; doi:10.1001/jamanetworkopen.2024.19640)
Supplement: Supplement 2. — Data Sharing Statement [file jamanetwopen-e2419640-s002.pdf]

Data Sharing Statement

Kaushik. In-Hospital Delirium and Disability and Cognitive Impairment After COVID-19 Hospitalization. *JAMA Netw Open*. Published July 02, 2024.
doi:10.1001/jamanetworkopen.2024.19640

Data
Data available: Yes
Data types: Deidentified participant data
How to access data: To request deidentified participant data from the VALIANT cohort or any of its studies, including the current study, please contact one of the VALIANT multiple PIs: Lauren Ferrante (lauren.ferrante@yale.edu), Andrew Cohen (andrew.b.cohen@yale.edu), or Alexandra Hajduk (alexandra.hadjuk@yale.edu).
When available: With publication

Supporting Documents
Document types: None

Additional Information
Who can access the data: The data will be made available to researchers whose proposed use of the data has been approved by the principal investigators.
Types of analyses: The data will be made available for research purposes and for other purposes approved by the principal investigators.
Mechanisms of data availability: A signed data use agreement (DUA) will be needed before the data can be shared.
